# The next phase in the development of ^13^C isotopically non-stationary metabolic flux analysis

**DOI:** 10.1093/jxb/erab292

**Published:** 2021-06-16

**Authors:** Thomas Wieloch

**Affiliations:** 1 Department of Medical Biochemistry and Biophysics, Umeå University, Umeå, Sweden; 2 Stellenbosch University, South Africa

**Keywords:** Carbon flux estimation, carbon metabolism, ^13^C labelling, ^13^CO_2_, complex models, ^13^C tracer experiments, labelling lag, metabolic flux analysis, model validation, photosynthesis

## Abstract

This viewpoint devises recommendations for future studies utilizing ^13^C isotopically non-stationary metabolic flux analysis to characterize plant metabolism. Most importantly, it highlights the necessity for model validation.

**^13^C isotopically non-stationary metabolic flux analysis (^13^C-INST-MFA) is an emerging technique for estimations of metabolic fluxes and pool sizes. Within the plant sciences, two studies utilizing this technique to characterize carbon metabolism have been published so far. Here, I examine these studies carefully. Readers unfamiliar with ^13^C-INST-MFA will obtain a critical understanding of the method and its findings. Readers working with ^13^C-INST-MFA are recommended to enter a phase of model validation to devise clear-cut protocols enabling robust estimations of specific fluxes.**

^13^C isotopically non-stationary metabolic flux analysis (^13^C-INST-MFA) is an emerging technique for estimations of metabolic fluxes and pool sizes. Within the plant sciences, two studies utilizing this technique to characterize carbon metabolism have been published so far. Here, I examine these studies carefully. Readers unfamiliar with ^13^C-INST-MFA will obtain a critical understanding of the method and its findings. Readers working with ^13^C-INST-MFA are recommended to enter a phase of model validation to devise clear-cut protocols enabling robust estimations of specific fluxes.

## Realistic reaction networks

**Fig. 1.**
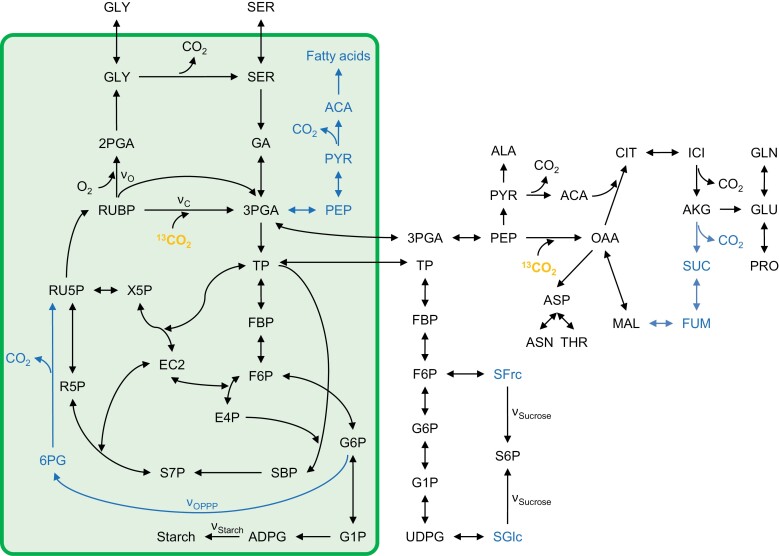
Reaction networks of published ^13^C-INST-MFA studies. Black: network as programmed by Ma *et al.* (2014; study 1) including reactions of the Calvin–Benson cycle, photorespiration, starch and sucrose biosynthesis, glycolysis, the tricarboxylic acid cycle, and amino acid biosynthesis. Blue: add-ons to the network of study 1 by Xu *et al.* (2021; study 2) including reactions of the oxidative pentose phosphate pathway, fatty acid biosynthesis, and the tricarboxylic acid cycle. Orange: ^13^C-enriched compound (label) entering the reaction network. Reactions inside the green box were programmed as chloroplast localized, while reactions outside the box were programmed as either cytosolic or without a compartment identifier. Abbreviations: 2PGA, 2-phosphoglycerate; 3PGA, 3-phosphoglycerate; 6PG, 6-phosphogluconate; ACA, acetyl-CoA; ADPG, ADP-glucose; AKG, α-ketoglutarate; ALA, alanine; ASN, asparagine; ASP, aspartate; CIT, citrate; E4P, erythrose 4-phosphate; EC2, enzyme-bound two-carbon fragment; F6P, fructose 6-phosphate; FBP, fructose 1,6-bisphosphate; FUM, fumarate; G1P, glucose 1-phosphate; G6P, glucose 6-phosphate; GA, glycerate; GLN, glutamine; GLU, glutamate; GLY, glycine; ICI, isocitrate; MAL, malate; OAA, oxaloacetate; PEP, phospho*enol*pyruvate; PRO, proline; PYR, pyruvate; R5P, ribose 5-phosphate; RU5P, ribulose 5-phosphate; RUBP, ribulose 1,5-bisphosphate; S6P, sucrose 6-phosphate; S7P, sedoheptulose 7-phosphate; SBP, sedoheptulose 1,7-bisphosphate; SER, serine; SFrc, fructosyl moiety of S6P; SGlc, glucosyl moiety of S6P; SUC, succinate; THR, threonine; TP; triose phosphate (glyceraldehyde 3-phosphate and dihydroxyacetone phosphate); UDPG, UDP-glucose; X5P, xylulose 5-phosphate; *ν*_C_, Rubisco carboxylation flux; *ν*_O_, Rubisco oxygenation flux; *ν*_OPPP_, OPPP flux; *ν*_Sucrose_, flux into sucrose; *ν*_Starch_, flux into starch.

In ^13^C-INST-MFA, a list of coded reactions specifies by which routes carbon can move from labelled or unlabelled sources through metabolic networks into sinks (Fig. 1). The reaction network of both studies allows direct export of 3-phosphoglycerate (3PGA) from chloroplasts to the cytosol. In the light, however, 3PGA export is believed to be restricted due to the chloroplast to cytosol pH gradient (Flügge *et al*., 1983, and references therein). Additionally, cytosolic reactions catalysed by glyceraldehyde-3-phosphate dehydrogenase and phosphoglycerate kinase are missing [conversion of glyceraldehyde 3-phosphate to 1,3-bisphosphoglycerate to 3PGA; triose phosphate (TP) to 3PGA]. Thus, ^13^C flux into glycolysis and the tricarboxylic acid cycle may follow unrealistic routes and has no cytosolic connection with sucrose biosynthesis. Furthermore, fractional refixation of respired CO_2_ is not considered (Loreto *et al.*, 1999), and numerous reversible reactions were programmed as irreversible, or *vice versa*. This includes reactions of the Calvin–Benson cycle catalysed by phosphoglycerate kinase and glyceraldehyde-3-phosphate dehydrogenase (conversion of 3PGA to 1,3-bisphosphoglycerate to glyceraldehyde 3-phosphate; 3PGA to TP), fructose bisphosphatase (conversion of fructose 1,6-bisphosphate to fructose 6-phosphate; FBP to F6P), and fructose-bisphosphate aldolase [conversion of dihydroxyacetone phosphate and erythrose 4-phosphate (E4P) to sedoheptulose 1,7-bisphosphate; TP and E4P to SBP]. Lastly, mesophyll chloroplasts reportedly lack enolase (van der Straeten *et al.*, 1991; Prabhakar *et al.*, 2009; Fukayama *et al.*, 2015). Thus, stromal conversion of 3PGA to phospho*enol*pyruvate (PEP) is likely to be infeasible, and fatty acid biosynthesis probably relies on PEP import from the cytosol. Future studies are encouraged to implement more realistic reaction networks representing carbon metabolism with all its intrinsic restrictions and freedom. Incorporation of cytosolic glyceraldehyde-3-phosphate dehydrogenases and phosphoglycerate kinase may enhance the utility of the model since these reactions proposedly constitute a central hub in leaf energy metabolism (Wieloch, 2021).

## Constrained fluxes

INCA allows users to constrain fluxes and pool sizes, for example based on independent physiological measurements or theoretical considerations. Users may specify constants or intervals, or choose not to impose any constraints. In all models (both studies), net CO_2_ assimilation was fixed at pre-determined values scaling fluxes up to reasonable values (supporting interpretation of results) while maintaining flux ratios. Additionally, study 1 fixed the absolute flux into starch (Fig. 1, *ν*_Starch_) and flux ratios between sucrose and amino acid biosynthesis according to physiological measurements. Similarly, study 2 fixed absolute fluxes into starch, sucrose (*ν*_Sucrose_), and amino acid biosynthesis, and the ratio of Rubisco oxygenation to carboxylation. This practice is potentially problematic since it may affect modelled flux ratios. Additionally, the necessity for constraints poses an important question. If large fluxes need to be fixed (*ν*_Starch_, *ν*_Sucrose_, and *ν*_O_/*ν*_C_), can one rely on ^13^C-INST-MFA to return credible results for unconstrained fluxes including smaller fluxes of interest (*ν*_OPPP_)? Thus, future studies are encouraged to present models without constraints alongside constrained models to show that data-driven flux estimation is feasible. Ideally, ^13^C data should drive the estimations with a minimum of imposed constraints.

## Effects of constrained fluxes on fluxes of interest

In principle, constraining fluxes or pool sizes can affect estimates of fluxes or pool sizes of interest due to interconnectivities within the reaction network (Fig. 1). In study 2, *ν*_O_/*ν*_C_ was constrained to be between 0.2 and 0.25. Modelling returned a *ν*_O_/*ν*_C_ ratio of 0.2, and an *R*_L_ and *ν*_OPPP_ of 5.2 μmol CO_2_ g^–1^ FW h^–1^ and 4.6 μmol CO_2_ g^–1^ FW h^–1^, respectively. When left unconstrained, modelling returned a physiologically unrealistic *ν*_O_/*ν*_C_ ratio of 0.09, and an *R*_L_ and *ν*_OPPP_ of 12.1 μmol CO_2_ g^–1^ FW h^–1^ and 10.5 μmol CO_2_ g^–1^ FW h^–1^, respectively. This indicates negative correlations between *ν*_O_/*ν*_C_ and *R*_L_ and *ν*_O_/*ν*_C_ and *ν*_OPPP_ (the lower photorespiration, the higher day respiration). Hence, fixing *ν*_O_/*ν*_C_ at values >0.2 may cause *R*_L_→0 and *ν*_OPPP_→0. Note that under normal growth conditions, *ν*_O_/*ν*_C_ ratios of 0.34 are common (Sharkey, 1988; Cegelski and Schaefer, 2006; Pärnik *et al*., 2007). Thus, future studies are encouraged to include sensitivity analyses investigating dependence between constrained fluxes and fluxes of interest.

## Validation of results by independent methods

INCA-based ^13^C-INST-MFA returns a comprehensive dataset containing estimates of (i) forward and reverse fluxes of all reactions and (ii) pool sizes of all metabolites specified in the reaction network (Fig. 1). Some of these items are accessible to other analytical techniques which, in principle, enables independent validation of ^13^C-INST-MFA results. Study 1 made no attempt to confirm modelled *ν*_O_/*ν*_C_ estimates by independent methods. However, estimated ratios were within the physiologically reasonable range. In contrast, study 2 tested the model estimate for *R*_L_ (5.2 μmol CO_2_ g^–1^ FW h^–1^) by the Laisk method which returned an *R*_L_ estimate of 9.3 μmol CO_2_ g^–1^ FW h^–1^ (Brooks and Farquhar, 1985). However, corresponding 95% confidence intervals showed no overlap (3.5–8.05 μmol CO_2_ g^–1^ FW h^–1^ versus 8.1–10.7 μmol CO_2_ g^–1^ FW h^–1^). Thus, these estimates are statistically different at the 0.05 significance level. Additionally, the model estimate for *ν*_OPPP_ in chloroplasts was compared with an estimate of flux through the cytosolic OPPP (Sharkey *et al.*, 2020). However, there is no reason to believe that these pathways carry the same flux. Thus, validation of estimates from ^13^C-INST-MFA by independent methods has not yet been achieved. However, independently determined fluxes currently used as constraints (*ν*_Starch_ and *ν*_Sucrose_) can be utilized to test the method by leaving them unconstrained and comparing modelled and measured values. Additionally, *ν*_O_/*ν*_C_ ratios may help to test the method since several alternative methods can provide independent estimates (Busch, 2013).

## Metabolically inactive pools or injection of carbon from unlabelled sources

**Fig. 2.**
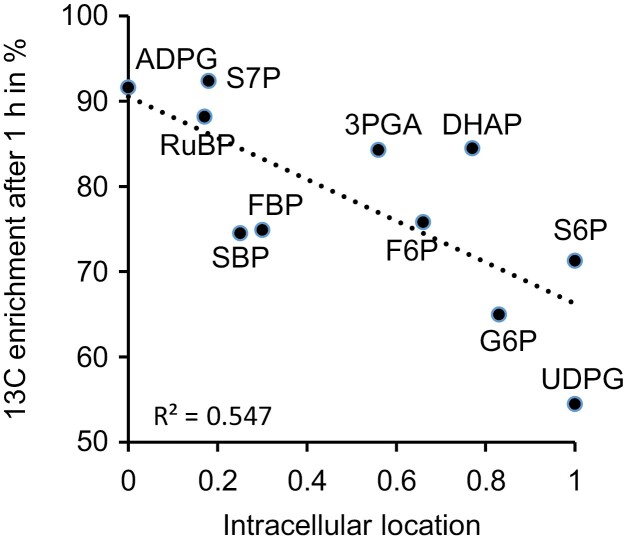
^13^C enrichment of metabolite pools in *Arabidopsis thaliana* rosettes 1 h into ^13^CO_2_ labelling as a function of the intracellular metabolite distribution from fully plastidial (*x*=0) to fully cytosolic (*x*=1). Abbreviations: 3PGA, 3-phosphoglycerate; ADPG, ADP-glucose; DHAP, dihydroxyacetone phosphate; F6P, fructose 6-phosphate; FBP, fructose 1,6-bisphosphate; G6P, glucose 6-phosphate; RuBP, ribulose 1,5-bisphosphate; S6P, sucrose 6-phosphate; S7P, sedoheptulose 7-phosphate; SBP, sedoheptulose 1,7-bisphosphate; UDPG, UDP-glucose. Re-analysed data from Szecowka *et al.* (2013). Sucrose and glucose 1-phosphate were excluded from the analysis since the former has a large vacuolar fraction and the latter reportedly exhibits an anomalous labelling behaviour (Szecowka *et al.*, 2013; Xu *et al.*, 2021).

In ^13^C-INST-MFA, ^12^C is progressively flushed out of the metabolic network and replaced by ^13^C from the labelling compound, such as ^13^CO_2_ (Fig. 1). Both studies reported fast initial labelling of metabolite pools. After several minutes, however, labelling slowed and, even after 1 h, a significant fraction of the pools remained unlabelled. This was attributed to metabolically inactive pools (i.e. metabolite pools disconnected from the flux of incoming ^13^C) and modelled accordingly by including a dilution term for each metabolite (accounting for apparently constant offsets between measured and modelled ^13^C enrichments). Alternatively, labelling lags may be explained in combination by breakdown of weakly labelled cytosolic sucrose into glucose and fructose, phosphorylation by hexokinase and fructokinase, and reinjection of glucose-6-phosphate-derived carbon into chloroplasts via a cytosolic OPPP not shown in Fig. 1 (Sharkey *et al.*, 2020). Figure 2 shows reported ^13^C enrichments of metabolites of the Calvin–Benson cycle, and starch and sucrose biosynthesis 1 h into ^13^CO_2_ labelling of *Arabidopsis thaliana* rosettes (Szecowka *et al.*, 2013). Additionally, these authors reported subcellular distributions of metabolites given on the *x*-axis from fully plastidial (*x*=0) to fully cytosolic (*x*=1). Interestingly, plastidial metabolites are more strongly ^13^C labelled than cytosolic metabolites. Metabolite distribution explains 55% of the labelling variability (*P*<0.01, *n*=11). Since it is not apparent why sizes of metabolically inactive pools would correlate with plastid–cytosol metabolite distribution, this corroborates the idea of injection of weakly labelled carbon into cytosolic metabolism. Future ^13^C-INST-MFA studies are encouraged to further explore this by expanding their reaction networks by sucrose breakdown pathways and a cytosolic OPPP. Additionally, sucrose, glucose, and fructose are large carbon pools with significant vacuolar contributions (Szecowka *et al.*, 2013). Thus, cytosol–vacuole transmembrane transport of these metabolites may need to be considered.

## Future focus

To date, evidence that ^13^C-INST-MFA returns reliable flux and pool size estimates is not available. Therefore, the field is recommended to enter a phase of validation of the complex models used in ^13^C-INST-MFA to devise clear-cut protocols enabling robust estimations of specific fluxes.

## Data Availability

The data supporting the findings of this study have been published by Ma *et al.* (2014), Szecowka *et al.* (2013), and Xu *et al.* (2021).

## Abbreviations

INCA: isotopomer network compartmental analysis
INST-MFA: isotopically non-stationary metabolic flux analysis
OPPP: oxidative pentose phosphate pathway
PEP: phospho*enol*pyruvate
3PGA: 3-phosphoglycerate
R_L_: day respiration
TP: triose phosphate
ν _O_/ν _C_: Rubisco oxygenation to carboxylation ratio
ν _OPPP_: OPPP flux
ν _Sucrose_: flux into sucrose
ν _Starch_: flux into starch
